# Studying the Size-Dependence of Graphene Nanoplatelets (GNPs) in the Final Properties of Polyurethane Aerogels: Thermal Insulation and Mechanical Strength

**DOI:** 10.3390/gels11010044

**Published:** 2025-01-07

**Authors:** Jaime Lledó, Judith Martín-de León, Tomás E. Gómez Álvarez-Arenas, Miguel Ángel Rodríguez-Pérez, Beatriz Merillas

**Affiliations:** 1 Cellular Materials Laboratory (CellMat), Condensed Matter Physics Department, Faculty of Science, Campus Miguel Delibes, University of Valladolid, Paseo de Belén 7, 47011 Valladolid, Spain; jaime.lledo@uva.es (J.L.); judit.martin.leon@uva.es (J.M.-d.L.); marrod@uva.es (M.Á.R.-P.); 2 BioEcoUVA Research Institute on Bioeconomy, University of Valladolid, 47011 Valladolid, Spain; 3 Departamento de Sensores y Sistemas Ultrasónicos (DSSU), Instituto de Tecnologías Físicas y de la Información (ITEFI), Consejo Superior de Investigaciones Científicas (CSIC), Serrano 144, 28006 Madrid, Spain; t.gomez@csic.es; 4 Department of Chemical Engineering, CERES, University of Coimbra, Rua Sílvio Lima, 3030-790 Coimbra, Portugal

**Keywords:** polyurethane/graphene nanoplatelet aerogel, thermal insulation, opacifiers, scattering, infrared radiation

## Abstract

In the present work, the influence of the addition of graphene nanoplatelets presenting different dimensions on polyurethane–polyisocyanurate aerogel structure and properties has been studied. The obtained aerogels synthesized through a sol–gel method have been fully characterized in terms of density, porosity, specific surface area, mechanical stiffness, thermal conductivity, and speed of sound. Opacified aerogels showing high porosity (>92%) and low densities (78–98 kg/m^3^) have been produced, and the effect of the size and content of graphene nanoplatelets has been studied. It has been observed that formulations with less than 5 wt.% of graphene nanoplatelets larger than 2 microns can effectively reduce the total thermal conductivity by absorption and scattering of the infrared radiation, reducing the heat transfer by this mechanism. The resulting opacified samples are highly insulating materials, with thermal conductivities less than 18 mW/m·K. Moreover, it has been observed that smaller particles with ca. 200 nm of average length can promote an increase in the elastic modulus, therefore obtaining stiffer aerogels, combined with thermal conductivities lower than 20 mW/m·K. Results have been studied in detail, providing a further understanding of the mechanisms for improving the final properties of these materials, making them more suitable for industrial applications.

## 1. Introduction

The interest in aerogels has been growing since their first synthesis in 1931 by S. Kistler [1]. The global aerogel market was calculated at more than a billion dollars in 2022 [2], and its rising trend is far from finishing. In fact, this market is estimated to reach 3.5 billion dollars in 2030 [2], making aerogels one of the most promising advanced materials worldwide. Their success can be attributable to the outstanding properties of these materials such as very low densities (3–350 kg/m^3^), extremely high specific surface areas up to 1000 m^2^/g, porosities higher than 80% [3], and excellent thermal insulation properties [4]. This singular combination of properties makes them ideal materials for some of the current leading sectors such as building, automotive, food packaging, aeronautic, or even aerospace [5].

Although having a huge range of applications, one of the most interesting and promising is their use as thermal insulators. Thanks to their unique porous structure, with pores in the nanoscale, and significantly low densities, aerogels may achieve very low thermal conductivities, reaching the superinsulation range, with values lower than 10 mW/m·K for silica aerogels [6]. However, silica aerogels are very brittle; thus, nowadays there are an important number of studies based on aerogels made from other materials, such as resorcinol–formaldehyde [7], cellulose [8,9], and polyurethane [10,11], which reach excellent thermal insulation while appeasing mechanical weaknesses. Polyurethane foams are one of the most used thermal insulators due to their low thermal conductivity in the range from 22 to 32 mW/m·K [12,13], versatility, and cost-effectiveness. Thus, polyurethane aerogels are a promising approach, as demonstrated by Merillas et al., reaching thermal conductivity values as low as silica aerogels, i.e., 12 mW/m·K [14].

To further improve the thermal insulation ability of these materials, a proper understanding of the heat transfer mechanism is essential. Heat transfer in porous materials is defined by the sum of four contributions: convection term (λ_c_), thermal conduction through the gas and solid phase (λ_g_ and λ_s_, respectively), and the radiation term (λ_r_). In materials with pores below 4 mm, air convection can be neglected [15,16], therefore, simplifying the equation.

Several strategies have been followed for reducing the thermal conductivity in aerogels, e.g., minimizing the intrinsic thermal conductivity of the solid phase, with materials such as resorcinol formaldehyde (0.18 W/m·K) [4], cellulose (0.04 W/m·K, depending on the manufacturing conditions) [17] or polyurethane (0.26 W/m·K) [18], or reducing the final pore size by controlling the synthesis so that Knudsen effect may occur [19].

Since the radiative term accounts for 30–40% of the thermal conductivity in aerogels and low-density porous polymers [20], reducing this term should have a significant impact on the final conductivity. When studying this term, it is important to consider the attenuation mechanisms involved: absorption by the aerogel matrix, which depends on the intrinsic radiation absorption of the solid phase, and scattering, related to the number and size of the scattering points, i.e., particles and pores in aerogels. The inclusion of infrared blockers is an effective method for reducing this term, taking advantage of the absorption and scattering processes of the radiation that these particles present [21,22]. Moreover, the inclusion of these fillers could lead to an improvement in mechanical properties [23,24], being an interesting approach to enhance the overall performance of these materials.

These infrared blockers, also known as opacifiers, could be divided into two big groups: mineral opacifiers, such as TiO_2_ and SiC nanoparticles; and carbon-based opacifiers, like carbon nanotubes (CNTs), graphene oxide (GO), or graphene nanoplatelets (GNPs). There exist some works that study the reduction in thermal conductivity by the inclusion of mineral opacifiers in silica aerogels [25,26,27,28].

Regarding carbon-based opacifiers, Lei et al. added GO nanosheets to monolith silica aerogels, reducing the thermal conductivity from 8.9 mW/m·K to 7.2 mW/m·K [6]. Liu et al. reduced the thermal conductivity from 25 mW/m·K to 18 mW/m·K on ambient pressure-dried silica aerogels by GO nanosheet addition [29]. Moreover, Lamy-Mendes et al. demonstrated that the inclusion of CNTs in polysilsesquioxane-based silica aerogel reduces the thermal conductivity from 36.8 mW/m·K to 31.2 mW/m·K [30]. A reduction in around 5 mW/m·K in thermal conductivity was also observed when adding 0.5 wt.% of GNPs with 0.19 µm of average size to polyimide aerogels [31]. Additionally, there exists a study where the thermal conductivity is increased by 16 mW/m·K when a percentage of 2% of GNPs are added to a polyvinyltrimethoxysilane-based aerogel, believed to be due to a growth in solid conductivity [24]. More recently, Zhu et al. studied different morphologies of opacifiers in silica aerogels, proving that there is an optimal number of protusions in TiO_2_ particles that lead to an optimized opacifying effect [32]. Then, Liu et al. developed a theoretical model for hollow opacifiers in silica aerogels in 2022, demonstrating that these hollow particles could present a more efficient opacifying effect than their solid counterpart [33]. In 2023, Deng et al. [34] prepared chitosan aerogels doped with hydroxyapatite nanowires, enhancing their mechanical properties by increasing their compressive elastic moduli up to five times, as well as maintaining their thermal insulation performance and improving their flame retardancy.

In polyurethane aerogels, the thermal conductivity has been reduced from 15 mW/m·K to 12 mW/m·K by the inclusion of a 3 wt.% of multiwalled CNTs [35]. Nevertheless, there are no studies of the influence of other opacifiers in this matrix and the effect of the blocker dimensions. The effect of the particle size is very important from a scientific perspective; the maximum radiation emitted by a blackbody is centered at around 10 μm [36]; therefore, particles with a comparable size to this wavelength may be able to scatter it through the well-known Mie scattering mechanism [37]. On the other hand, if particles are smaller than one-tenth of this wavelength, the Rayleigh scattering mechanism may arise [38], and the scattering process of infrared radiation will be weaker. Thus, the size of the infrared blockers plays a main role in the subsequent increase in the extinction coefficient.

Therefore, herein polyurethane aerogels containing different contents of 2-D GNPs with dimensional differences (length and thickness) have been synthesized and their final properties have been studied in detail. The influence of the opacifiers in the aerogel porous structure, textural properties, mechanical properties, and thermal conductivity are analyzed and compared with the non-doped aerogel. Moreover, a study of the contributions to the total thermal conductivity has been performed by analyzing each of the heat transfer mechanisms; solid conduction, gas conduction, and radiation, seeking for the maximum reduction in the radiation term without significantly modifying the solid and gaseous contributions. Despite other opacifiers have been already used in the literature, the study of the influence of their size to promote an effective scattering of radiation is relevant information for reaching the production of superinsulating materials. Thus, the main objective of this work is to expand the knowledge on the influence of opacifiers when being included in polyurethane aerogels and analyze the effect on their final properties. Thus, the aim is to expand their promising industrial applicability, as well as providing further knowledge on the key characteristics that IR blockers should have to maximize their effect. In addition, it is remarkable that the strategy proposed in this paper could provide relevant insights for other thermal insulators, a very important task given the current requirements for developing better thermal insulating materials.

## 2. Results and Discussion

### 2.1. Graphene Nanoplatelets Characterization

**Figure 1:**
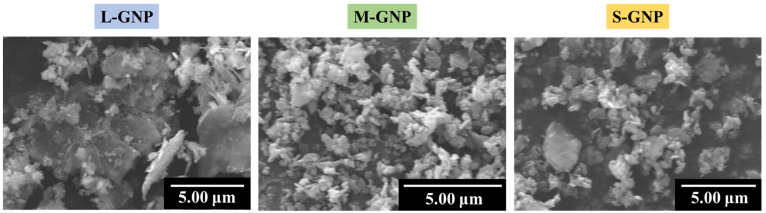
SEM micrographs of the GNPs used in the present work.

Figure 1 displays the SEM micrographs of the different GNP fillers used in this work, where the differences in lateral size can be appreciated.

Three GNP types with different sizes have been used in this study and are labeled according to their dimensions in the following way: Plat-7 as Large, Plat-2 as Medium, and Nano-307 as Small. Therefore, samples are labeled as X-Y, where X is the filler content and Y is the filler type. An example of nomenclature of the opacified aerogels could be 0.5 L, being that the opacified aerogel with 0.5 wt.% of Plat-7 GNP. It is possible to observe the difference in size between the GNP, leading to differences in the specific surface area.

### 2.2. Aerogel’s Characterization

#### 2.2.1. Gelation Kinetics and Chemical Bonding

The gelation time for the reference gel is 4 min. A reduction in the gelation time was generally observed when adding the different fillers as seen in Table 1, suggesting that the GNP presence favors the formation of the final gel, depending on the content and type of the GNP used. This could be explained by the favored nucleation and growth of the particles formed during the polymerization reactions when GNPs are present in the solution.

The reduction in gel time is consistent with favored nucleation when GNPs are present, confirmed through the FT-IR spectra of all the samples, showing an increase in the peak area (see Figure S1 in Supporting Information for more details).

#### 2.2.2. Density and Shrinkage

**Figure 2:**
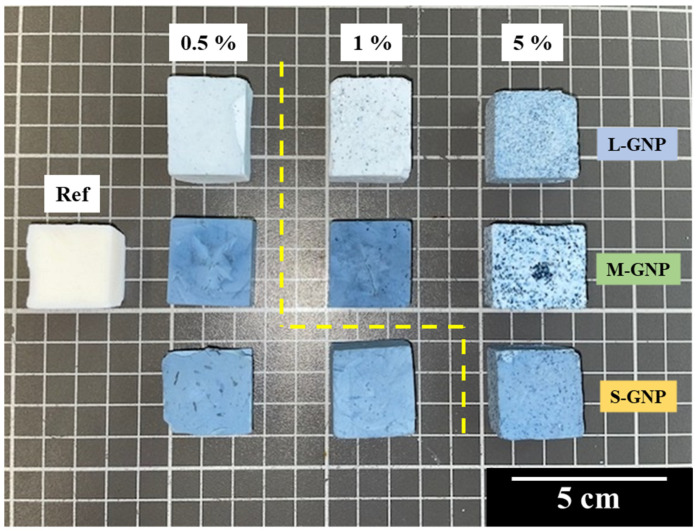
Photo of all the synthesized aerogels in this work. Aerogels with the same type of GNP are placed in the same row, while in the same column are sorted by GNP content. The dashed yellow line indicates when the particle aggregates start to be visible.

Figure 2 shows all the synthesized aerogels in this work following the method described in Section 4.2.

The addition of these GNPs makes the aerogels bluish, making the intensity of the final color greater for larger opacifier contents. Despite the initial solution being sonicated, some aggregates can be seen when the content of GNP grows, which causes a decrease in the color intensity in samples with higher GNP content, e.g., 5 M. Regarding these visible aggregates, a clear dependence is observed with the GNP size, since the smallest GNP (S-GNP) does not form them until 5 wt.% content. However, in the case of the large and medium-sized particles (L-GNPs and M-GNPs), the visible aggregates are seen from a 1 wt.% amount.

Table 2 collects data from L-GNP, M-GNP, and S-GNP series. A total of nine different opacified aerogels have been synthesized, which are compared with the non-opacified aerogel as reference.

**Figure 3:**
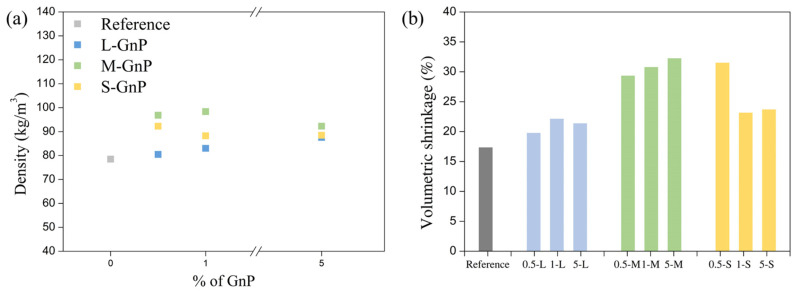
(**a**) Densities of the opacified samples and the reference one and (**b**) volumetric shrinkage (S_v_) for all the produced materials.

The data shown in Table 2 is discussed in this section and the following ones. Figure 3 shows the tendencies regarding density and volume shrinkage. It is observed a slight growth in the final density in comparison with the reference aerogel (Figure 3a). While the reference aerogel shows a value of 78.47 kg/m^3^, the opacified samples present densities in the range of 80–98 kg/m^3^, being the M-GNP ones that provide the highest density samples, followed by the S-GNP. On the other hand, the densities of the L-GNP samples are the ones closest to the reference value. Density results can be attributed to the added mass of GNP and changes in the linear and volumetric shrinkage experimented during drying, which causes slight compaction of the whole structure. As observed in Figure 3b., M-GNP and S-GNP samples have the larger volumetric shrinkage values, reaching ca. 30% in some samples, being the ones with the highest densities, whereas all values for the L-GNP samples are below 26% and close to the reference sample, explaining their lower density. In any case, the porosity values are still large and common for PU aerogels, since all of the samples have porosities over 92%.

#### 2.2.3. Nanoporous Structure

**Figure 4:**
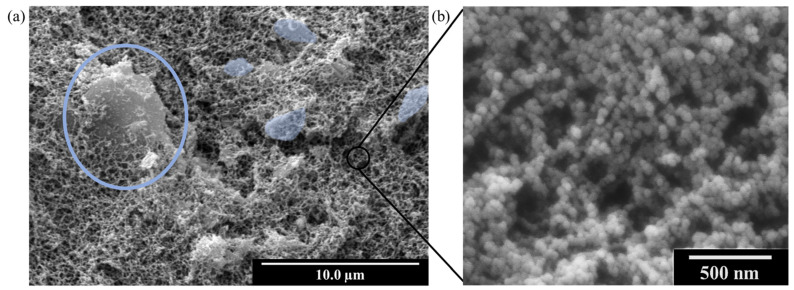
(**a**) SEM micrograph of the 5 L opacified aerogel. L-GNP particles are marked in blue color, surrounded by the pearl-necklace aerogel structure. (**b**) Magnification of the aerogel nanostructure.

As discussed in Section 2.2.2, the produced materials present some aggregates visible to the naked eye (Figure 2). However, the whole internal structure of the final materials is highly homogeneous since the majority of the GNPs are well dispersed in the aerogel matrix (see Figure S2). Figure 4 shows some SEM micrographs of the 5 L sample. It can be seen how GNPs are embedded within the aerogel structure, while the aerogel particles grow homogeneously around the nanoplatelets. The micrographs for the rest of the samples are included in the Supporting Information (Figure S3).

**Figure 5:**
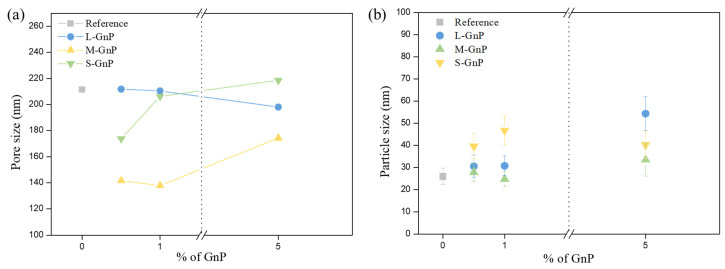
(**a**) pore size and (**b**) particle size of the opacified samples and the reference one. The dotted lines and the axis-break are visual guides to improve the visualization of data.

Regarding the specific surface area, Table 2 shows that all the samples present a value in the range between 192 and 272 m^2^/g. With respect to the aerogel pore volume, the obtained values are similar, with slight differences caused by the addition of GNP due to the volume occupied by these fillers, and the increased shrinkage as mentioned in the previous section. This slight reduction in the final pore volume while observing the differences among specific surface areas, leads to pore sizes that vary between 137 and 218 nm (Table 2). This behavior is also related to the shrinkage values previously commented, being the samples with higher shrinkages are those with smaller pore sizes. Hence, M-GNP samples show the smallest pores, while larger pores are found in S and L-GNP series (Figure 5a).

The particle size of these materials has been measured by the method described in Section 4.3.5. It is observed that the particle size slightly increases when adding GNPs (Figure 5b). This effect is compatible with the reduction in gelation time, which can also be observed in formulations with higher amounts of catalyst [39].

### 2.3. Mechanical Properties

#### 2.3.1. Compression–Decompression Cycles

**Figure 6:**
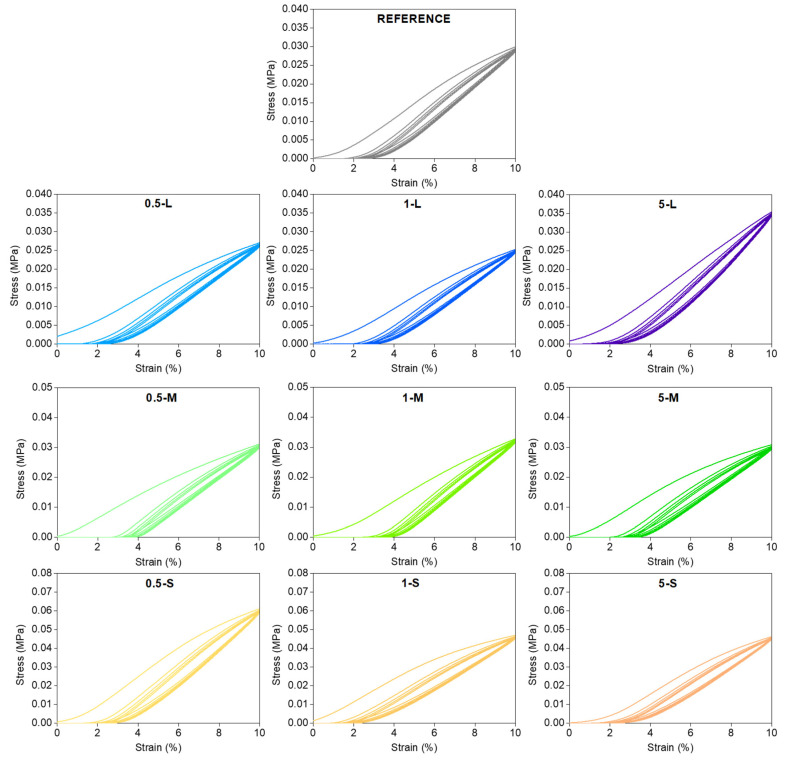
Stress–strain curves for the opacified aerogels and the reference one obtained by compression-decompression tests up to a 10% deformation.

Figure 6 shows the compression–decompression cycles of all the samples. It can be observed that all of them show a remarkably similar shape, with an almost complete recovery after the five cycles (non-recoverable deformation of approximately 2%). This shows that all the aerogels studied in this work have a significant elastic behavior, with high recovery ratios after being compressed to a 10% strain.

**Figure 7:**
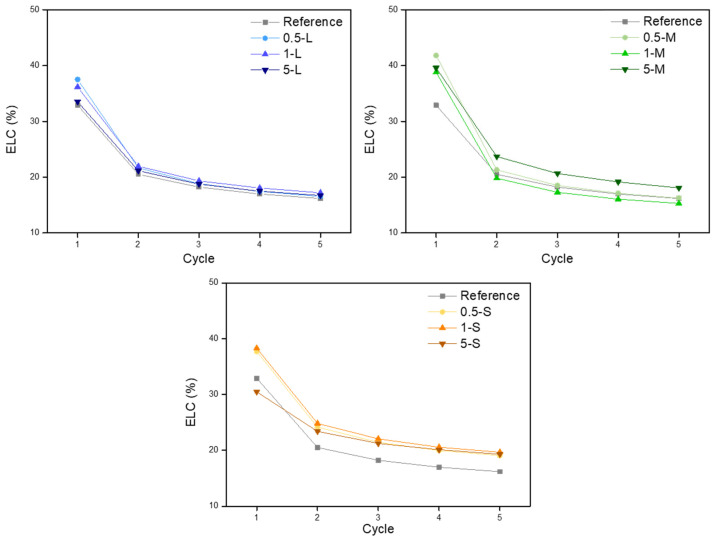
Energy loss coefficients for each GNP size and content.

The energy loss coefficients were calculated from the hysteresis area of the compression-decompression cycles, following Equation (10). This parameter gives information about the elasticity of the material, being lower for samples presenting more elastic behavior. The obtained values for this parameter are shown in Figure 7.

**Figure 8:**
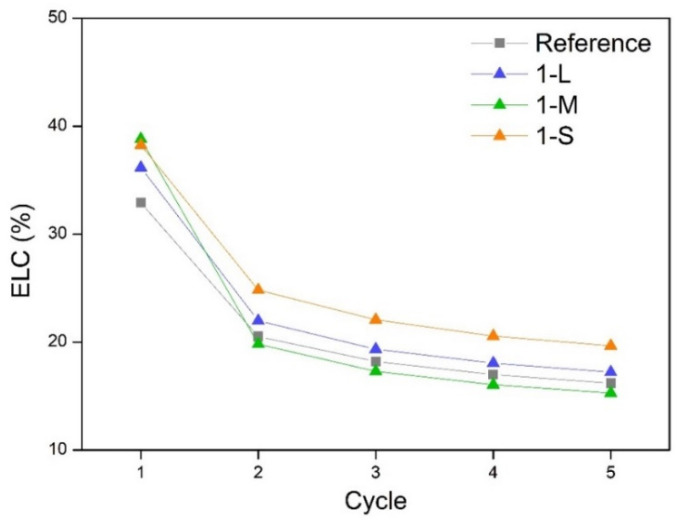
Comparison of the ELC for samples with different GNPs with the same content (1 wt.%).

In all cases, the ELC was higher in the first cycle than in the others, meaning that a larger amount of energy was dissipated. However, as this coefficient decreases when the rest of the cycles are performed, the elasticity of the materials grows, reaching lower ELC values when using larger particles such as L and M-GNP, while the ELC values are higher when using S-GNP particles, thus obtaining fewer elastic materials (Figure 7 and Figure 8). All these data indicate that S-GNP fillers reduce the elasticity to a higher extent than the larger ones used in this study. This could be related to the better dispersion reached when using these fillers of a smaller size.

#### 2.3.2. Elastic Modulus

**Figure 9:**
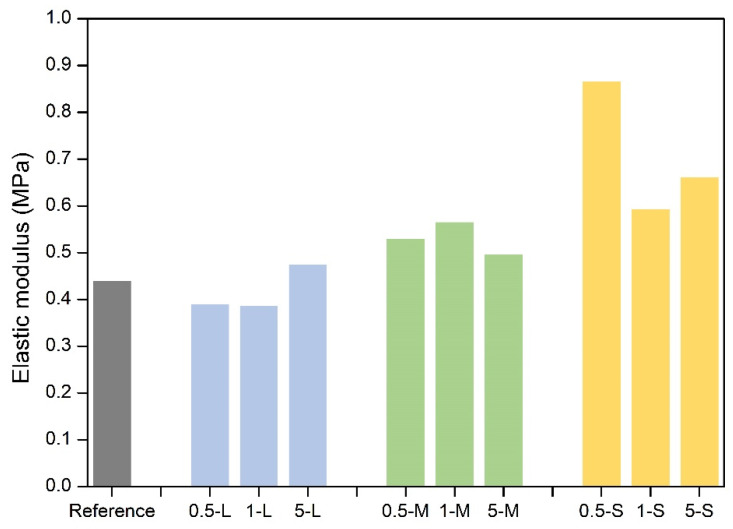
Raw values of elastic modulus of all the samples.

The elastic modulus values of all the samples are plotted in Figure 9. The reference aerogel has an elastic modulus of 0.44 MPa, which is a higher value than most of the silica aerogels with similar densities (0.022–0.1 MPa [30]), showing a better mechanical performance than these ones.

**Figure 10:**
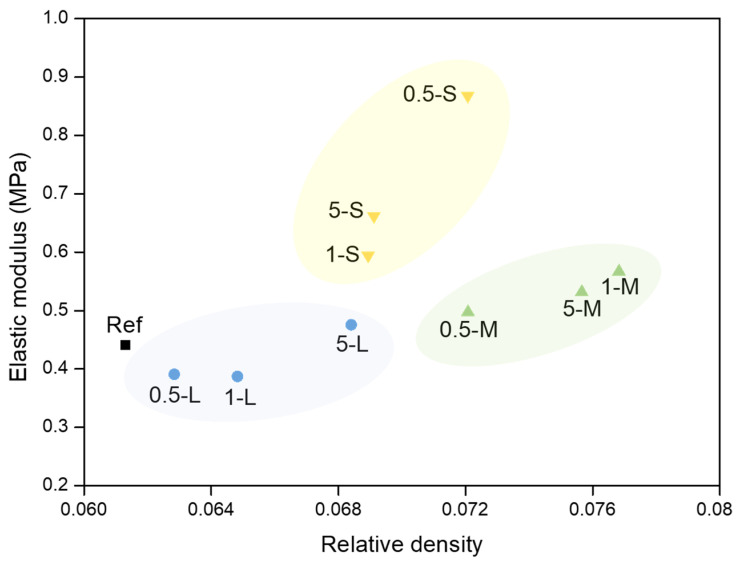
Elastic modulus vs. relative density of all the samples. L-GNP series in blue, M-GNP series in green, and S-GNP series in yellow.

It is observed that S-GNP samples provide the largest elastic modulus values, whereas the values of the L and M-GNP series are similar to those of the reference. To study the actual influence of these particles in the opacified aerogels mechanical stiffness, the dependence of the elastic modulus on the density must be investigated. In Figure 10, the elastic modulus versus the relative density is plotted.

It is noticeable that the relative density growth in the L and M-GNP series slightly affects the elastic modulus, with values close to the reference one and a small increase as density grows. However, a significant growth in the elastic modulus is observed in the S-GNP series both, in comparison to the reference, and with density. This increase cannot be solely explained by the growth of the relative density, as this is not observed in the L and M-GNP series. Therefore, the addition of S-GNP seems to have an additional effect on the aerogels structure that improves the stiffness of these materials. This effect in the aerogel stiffness can be related to the higher number of particles included in the final aerogel with S-GNP, owing to the smaller size, in combination with a better dispersion throughout the aerogel structure that provides a strong effect on their mechanical resistance.

**Figure 11:**
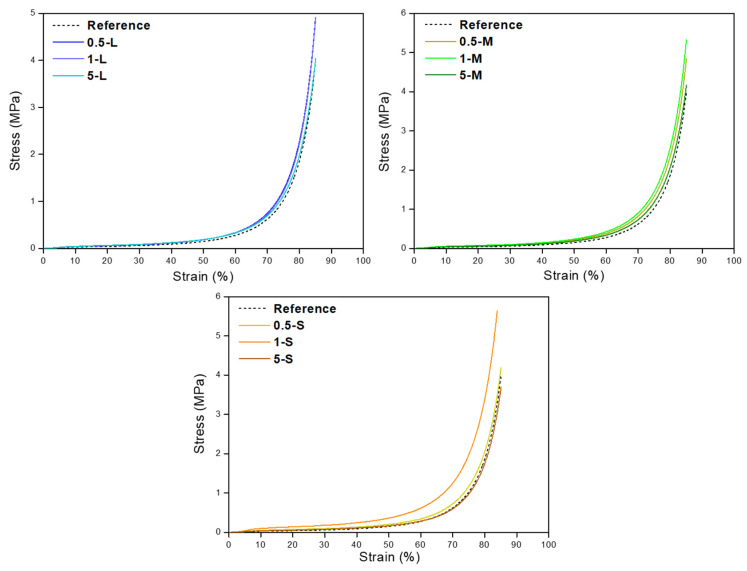
Stress–strain curves until a deformation of at least 80% of all the samples, separated by GNP type. In the three graphs, the dashed line represents the reference aerogel curve.

The strain–stress curves up to 80% strain are shown in Figure 11. It is noticeable that all the samples reach more than 80% strain without showing any breaking point. Except for the 5 S sample, every material requires higher stress than the reference one to reach that deformation, obtaining, in general, mechanically more resistant aerogels in comparison with the reference one.

Therefore, the inclusion of S-GNPs, led to a significant improvement in the mechanical stiffness of the reference polyurethane aerogel, especially at low contents of 0.5 wt.%, increasing the elastic modulus from 0.44 MPa to ca. 0.90 MPa. The inclusion of nanoparticles in order to enhance the stiffness of aerogels is proven to be an effective strategy not only in polyurethane aerogels but also in aerogels based on different matrixes [34].

### 2.4. Thermal Conductivity Analysis

**Figure 12:**
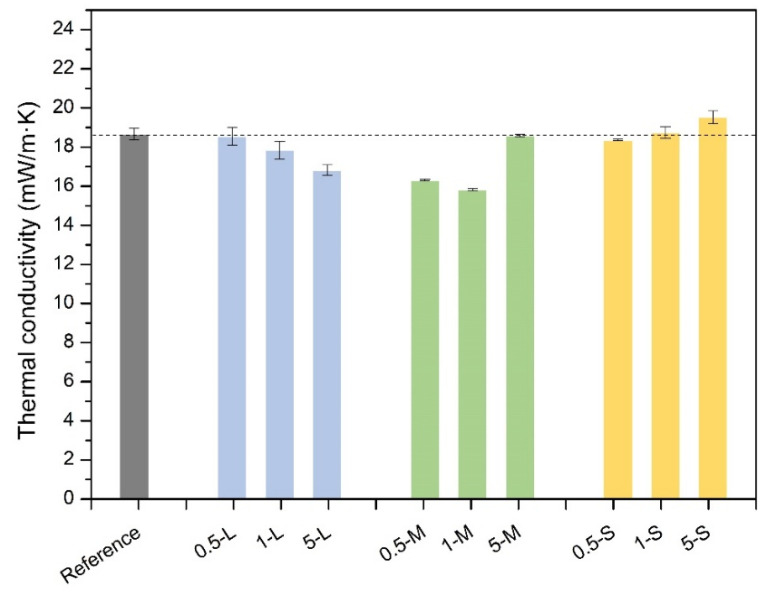
Thermal conductivity of the studied aerogels at 20 °C. The gray bar represents the reference aerogel (non-opacified). For the sake of clarity, the dashed line represents the thermal conductivity of the reference aerogel.

The thermal conductivity of all the synthesized aerogels has been measured by the method described in Section 4.3.7. The obtained values are plotted in Figure 12, and values of the thermal conductivities at different temperatures can be found in Figure S4 of Supporting Information. Out of nine opacified samples, six of them have a lower thermal conductivity than that of the reference aerogel, all of them in the range of superinsulating materials (below that of the air, i.e., 26 mW/mK). Moreover, it is possible to observe different behaviors and tendencies depending on the GNP size. The thermal conductivity of the reference material has been reduced by 0.5%, 4%, and 10% for the samples 0.5 L, 1 L, and 5 L, respectively, whereas when using M-GnP the reduction reaches almost 13% in the 0.5 M sample and 15% in 1 M.

Finally, in the S-GNP series, the thermal conductivity has not been reduced in fact, it has been even increased in the case of 1 S and 5 S samples. Thus, L and M-GNPs are effective fillers to reduce the thermal conductivity of aerogels, while particles of the same nature but a smaller size (S-GNP) cannot effectively reduce the thermal conductivity.

To understand the experimented improvement in the thermal insulation performance of the L and M aerogel series, it is necessary to study each of the contributions to the total thermal conductivity. As previously explained, the thermal conductivity of aerogels can be described by the sum of the conduction through the gaseous and solid phases, and a radiative term. Conduction through the solid phase can be calculated with Equation (1):

(1) $$\lambda_{s}=\rho_{r}\cdot \lambda_{s}^{\prime }\cdot \frac{\nu }{\nu_{s}}$$

where ρ_r_ is the relative density, $\lambda_{s}^{\prime }$ is the thermal conductivity of the solid matrix (0.26 W/m·K [18]), ν is the sound speed through the aerogel, and ν_0_ the sound speed of the solid matrix, being 1710 m/s in the case of polyurethane [4]. (Sound of speed measurements of all the aerogels can be found in Table S1).

Regarding the thermal conduction through the gaseous phase, it is important to consider the Knudsen effect, which sharply reduces this term when the pores are in the nanometric range. This contribution can be calculated with Equation (2):

(2) $$\lambda_{g}=\left(1-\rho_{r}\right)\cdot \lambda_{g}^{\prime }=\left(1-\rho_{r}\right)\cdot \frac{\lambda_{g0}^{\prime }(T)}{1+\frac{2\beta l_{g}}{\Phi_{pore}}}$$

where $\lambda_{g0}^{\prime }$ is the thermal conductivity for the gas inside the pores of the material, and l_g_ is the mean free path of the gas molecules (c.a. 70 nm for air [40,41]), β is a correlation factor with a value of 1.64 for air [35], and φ_pore_ is the average pore size.

The solid and gaseous conduction contributions were calculated with Equations (1) and (2), respectively. Nevertheless, the radiative term was calculated by subtraction of both terms from the measured thermal conductivity in order to evaluate the impact of the opacifiers’ inclusion in the final extinction coefficient of the aerogel samples.

**Figure 13:**
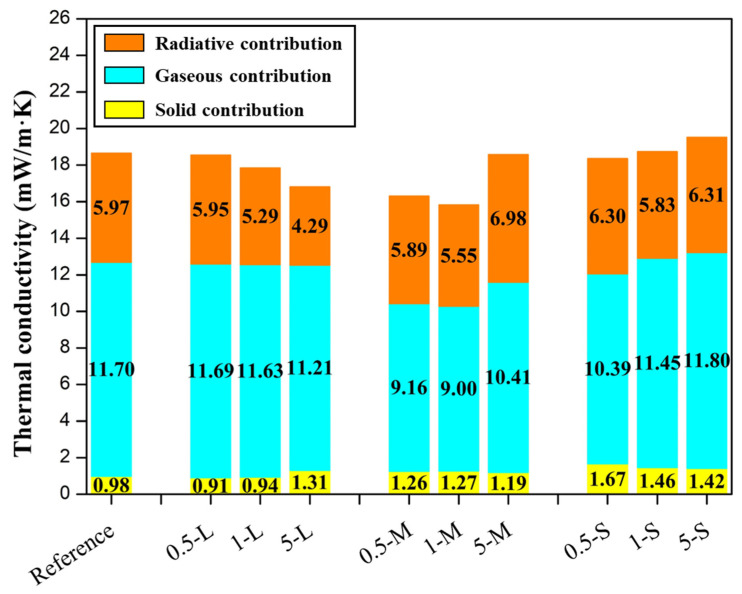
Contributions to the total thermal conductivity of opacified aerogels.

In Figure 13, the contributions to the total thermal conductivity of all the samples from this work are shown.

The contribution with the highest weight is the conduction through the gaseous phase since it accounts for between 56% to almost 67% of the total thermal conductivity. As previously analyzed, the addition of GNPs to these materials has a direct influence on their nanostructure, observing that a smaller pore size leads to a lower gaseous thermal contribution. Thus, the gaseous contribution follows the same tendency as pore size (Figure 5a), presenting lower values for the samples with lower pore sizes as 0.5 M and 1 M, while in 5 M this value increases as pores are larger in this sample. Smaller pores in the 0.5 S sample also led to smaller gaseous contributions, while it becomes larger as the content of S-GNP increases, i.e., 1 S and 5 S samples. However, in the L-GNP series, the pore size does not significantly vary with respect to the reference; thus, the gas conduction values are similar to the reference value.

The solid contribution is intrinsically low, being comprised between 0.91 and 1.67 mW/mK. In the L-GNP series, the solid contribution is very similar to the reference one, being slightly higher in the 5 L sample, owing to the large amount of added fillers. For the M-GNP series, this contribution is higher than when using larger GNP, probably related to smaller GNP size and, in comparison with the L-GNP series, higher density values, which also contributes to enhancing this term. Finally, an increase in this term is observed when lowering the size of the GNP used, revealing that the phonon transfer is favored in formulations with smaller GNP, likely due to their better dispersion in the polymer matrix that creates a more homogeneous path. Thus, in the S-GNP series, an increase in this term with respect to the reference one is observed, as smaller GNPs have been used, and slightly higher values of density are present. This demonstrates that GNP size is highly related to solid conduction in these materials, being higher as smaller fillers are used, even though some differences in density are present. The addition of these fillers could increase the solid conduction since they may act as “connectors’’ between the particulate skeleton, enhancing the phonon transfer. These percolation networks are more likely to form when the particles added are smaller; therefore, the number of particles to reach a certain percentage is higher. In fact, the number of particles can be estimated with the size of these fillers, being almost 5000 times higher when using S-GNPs than when using the same weight percentage of L-GNP, which supports the explanation of the growth in this term related to GNP size.

**Figure 14:**
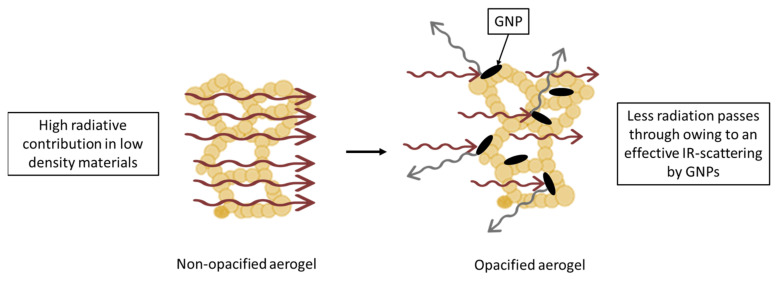
Diagram of the scattering mechanism in opacified aerogels with large GNPs (L-GNPs and M-GNPs).

The radiative term also accounts for a considerable percentage (ca. 30%) of the total thermal conductivity. It is observed that the addition of GNPs to these aerogels reduces this term from 5.97 mW/m·K value from the reference aerogel to 4.29 mW/m·K in the 5 L formulation, the sample with the highest reduction in the radiative term, implying a reduction in a 28% in this term and a 10% with respect to the total thermal conductivity, verifying the absorbing and scattering-based explanation (Figure 14). On the other hand, in the S-GNP series, there is not a reduction in radiative contribution probably due to that these particles are not big enough to scatter the infrared radiation, and the absorption process that these particles present due to their nature could not be enough to effectively reduce this term. In fact, this radiative term is slightly higher than in the reference sample. In the case of M-GNP, the 0.5 and 1 M samples show lower radiation contributions, while in the 5 M sample, this term is higher.

Despite the effects on the porous structures and their influence on the gaseous and solid conductions, the reduction in the radiative term when using graphene nanoplatelets with an appropriate size for the scattering effect of infrared radiation is clear and significant, as previously reported in other works [22]. This can be explained by the black-body thermal radiation emitted by an object, centered at around 10 μm [36]; thus, opacifiers around this size will effectively scatter the infrared radiation, reducing their contribution to the heat transfer, while smaller particles will not be able, as seen for S-GNP, revealing that the absorption effect of these carbon-based fillers may not be enough to block the infrared radiation. The optimum size for blocking the infrared radiation is reported to be 4 μm for mineral opacifiers such as SiC [42]. This work quantifies the optimum size for carbon-based opacifiers with platelet shape: particles with a lateral size between 2 and 7 μm can induce an effective reduction in the radiative term by scattering the infrared radiation in aerogels, making it possible to extend this to other porous materials.

The overall performance of these aerogels should be compared with traditional insulating materials such as polypropylene, polyamide, or polyurethane foams, considering both mechanical performance and thermal insulating properties. In general terms, foams present higher elastic modulus than the aerogels presented in this work (0.38–0.86 MPa), with values of 1.4 MPa for polyamide foams with densities around 100 kg/m^3^ [43], 1.8 MPa for neat polypropylene foam with a relative density of 0.2 [44], or even 4.4 MPa for rigid polyurethane foams with lower densities of ca. 30 kg/m^3^ [45]. However, the thermal conductivity of these foams is higher than the ones presented by the PU/GNP aerogels presented herein. These aerogels reach reductions between 20 and 46% when compared with typical polyurethane foams [46], and up to 55%, when compared with PP foams [47]. Moreover, PU/GNP aerogels can also be compared with silica aerogels, which may present thermal conductivities as low as 12 mW/m·K [48], whereas their elastic modulus reaches lower values, as commented in Section 2.3.2, proving the excellent overall properties that the materials herein presented show. This excellent thermal insulation behavior may compensate for the lower elastic modulus in comparison to traditional thermal insulation materials, making them materials with an interesting combination of properties suitable from an industrial perspective.

**Figure 15:**
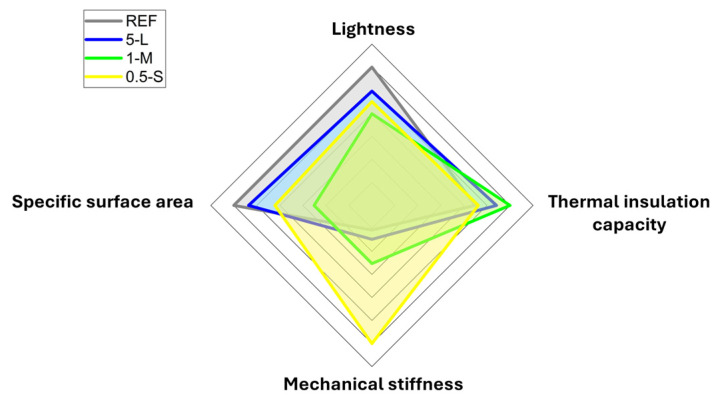
Diagram of four of the most important parameters of aerogels from the reference, 5 L, 1 M, and 0.5 S samples.

Finally, in Figure 15, the most important parameters of aerogels are depicted, in which lightness is related to the materials’ density, the thermal insulation capacity is related to the thermal conductivity, and the mechanical stiffness is related to the elastic modulus, and, finally, the specific surface area. In this graph, it can be observed how each parameter varies depending on the particle added, observing different trends for each particle size. It can be concluded that the 1 M sample presents the highest thermal insulation capacity, i.e., the lowest thermal conductivity, while its lightness and specific surface area are lower in comparison with the other samples. On the other hand, the 0.5 S sample presents the highest mechanical strength, while being denser. This graph can visually help to find the optimal sample depending on the final properties of a desired application.

## 3. Conclusions

The effect of the addition of graphene nanoplatelets in polyurethane aerogels has been studied for the first time, observing a size dependence on the final properties of the opacified samples. In terms of thermal conductivity, six out of nine samples showed reduced thermal conductivity in comparison with the non-opacified sample, all of them in the range of super-insulating materials. Large (L-GNP) and medium (M-GNP) particles were shown to be effective fillers for reducing the thermal conductivity due to their optimum particle size that efficiently promotes the scattering of radiation that, in combination with the intrinsic absorption process, minimizes the amount of radiation passing through these materials. Nevertheless, the smallest particles (S-GNP series) did not show an improvement in the insulation properties, although they reached an increase in the mechanical stiffness acting as reinforcing points.

The opacified polyurethane aerogels presented densities ranging between 80 and 98 kg/m^3^, pore sizes between 137 and 220 nm, and particles with a size between 25 and 55 nm. These differences are caused by the inclusion of GNP, observing an increase in density and particle size. On the other hand, the pore size is reduced with the inclusion of these fillers, in particular for the medium particles, M-GNP.

The mechanical properties have been analyzed by compression tests. S-GNPs lead to a two-fold increase in the mechanical stiffness of the samples, being less elastic than the rest of the studied aerogels. Thus, the addition of S-GNP particles to PU-PIR aerogels seems to be an effective methodology to improve the stiffness of these materials.

A significant reduction in thermal conductivity has been observed when using GNPs bigger than 2 microns of lateral size by an opacifying effect, based on simultaneous scattering and absorption mechanisms, since the thermal conductivity is not reduced when using smaller particles (S-GNP). L-GNP particles are the best option for reducing the thermal conductivity, reducing the radiative term up to 30%, due to the higher scattering of infrared light that they present thanks to their bigger size, combined with the absorption process that takes place thanks to their chemical nature.

This work broadens the knowledge of how to tailor the properties of polyurethane aerogels with graphene nanoplatelets as fillers, making it possible to adapt the amount and size of fillers in these materials depending on the desired application. The use of these materials in thermal insulation and energy-related applications is improved in two ways, reaching lower thermal conductivity values, or obtaining stiffer materials without losing the super-insulating characteristics, making them more appropriate for potential industrial applications in both cases.

## 4. Materials and Methods

### 4.1. Materials

IsoPMDI 92140 (ρ = 1.23 g/cm^3^ at 25 °C) corresponding to the formulation 4,4′-diphenylmethane diisocyanate was provided by BASF Española S.L (Barcelona, Spain). The polyol used was pentaerythritol (purity > 98%) purchased from Alfa Aesar (Thermo Fisher GmbH, Kandel, Germany). Kosmos 75 MEG was employed as a catalyst, and it was kindly supplied by Evonik (Essen, Germany).

Scharlab, S.L. (Barcelona, Spain) supplied the following solvents: acetone (technical grade), acetonitrile (HPLC grade), tetrahydrofuran stabilized with 250 ppm BHT, and dimethyl sulfoxide (purity > 99%).

Plat-7 graphene nanoplatelets (GNP) (lateral size = 7.0 μm, thickness = 2.5 nm, specific surface area = 70 m^2^/g) and Plat-2 GNPs (lateral size = 2.0 μm, thickness = 10 nm, specific surface area = 200 m^2^/g) were purchased from Avanzare Innovación Tecnológica S.L (La Rioja, Spain). Nano-307 GNPs (lateral size = 100 nm, thickness = 2.5 nm, specific surface area = 350 m^2^/g [36]) were provided by Asbury Carbons Inc. (Asbury, NJ, USA). Both solvents and GNPs were used without further purification or modification.

### 4.2. Synthesis of Aerogels

Polyurethane–polyisocyanurate (PU-PIR) aerogels were synthesized by the previously described procedure by Merillas et al. [14]. Different contents of GNPs (0.5, 1, and 5 wt.% with respect to the sum of polyol and isocyanate mass) were added to the isocyanate solution (44 g/L in a mixture of 65:35 ACN/THF). This solution was firstly sonicated in a water bath for 10 min (increased to 20 min in the case of 5 wt.% content) to improve the filler dispersion and reduce the formation of GNP aggregates. Ice was added to the bath in order to avoid temperature rising during the sonication. Then, a certain amount of polyol solution (100 g/L of pentaerythritol in DMSO) was added to the isocyanate and, immediately after, a calculated amount of catalyst (70 g/L in THF) was added so that the content of catalyst in the final mixture was 4 wt.% over the sum of the polyol and isocyanate mass. Once the components were added, the solution was mixed with a magnetic stirrer at 300 rpm until the gel formed. When the solution viscosity was high, the magnetic stirrer was quickly removed from the solution to avoid defects in the final gel. The transition from sol to gel is noticeable due to the high growth in the viscosity and change in color from the black liquid sol to the bluish final gel.

Once the gel was formed, it was covered with acetonitrile and aged for 24 h. When the aging step was over, the gels were washed twice each 12 h with clean acetonitrile. Finally, these gels were dried by supercritical drying at 45 °C and 120 bar.

### 4.3. Aerogels Characterization

#### 4.3.1. Gelation Time

Gelation time is defined as the time from when the reaction starts until the gel does not flow anymore by tilting the container. It can be detected due to a change in the color of the solution and a strong increase in the final viscosity. As accurate determination of this point may be difficult, the gel time is defined as the time that the magnetic stirrer is taken out from the solution. This time was monitored with a stopwatch.

#### 4.3.2. Shrinkage

Shrinkage was studied by comparing the size of the aerogels before and after the drying step. Linear shrinkage (S_L_) and volumetric shrinkage (S_V_) were calculated with the following Equations (3) and (4):

(3) $$S_{L}\;(\%)=\left(1-\frac{d}{d_{0}}\right)*100$$

(4) $$S_{V}\;(\%)=\left(1-\frac{V}{V_{0}}\right)*100$$

where d_0_ is the diameter of the wet gel before drying, d is the diameter of the final aerogel, and V_0_ and V are the initial and final volumes.

#### 4.3.3. Density and Porosity

Bulk density (geometrical density) is obtained with Equation (5) by dividing the mass of the aerogel by the occupied volume:

(5) $$\rho_{B}=\frac{m}{V}$$

This method is described by ASTM D1622/D1622M-14 [49]. The mass was measured with an AT261 Mettler Toledo balance, and the volume was calculated by measuring the monolith dimensions with a caliper.

The relative density (ρ_r_) is calculated as the fraction between the bulk density and the solid density, following Equation (6):

(6) $$\rho_{r}=\frac{\rho_{B}}{\rho_{s}}$$

where $\rho_{s}$ is the solid density of polyurethane, being 1.28 g/cm^3^, the mean value of the synthesized samples measured by helium pycnometry.

Finally, porosity (Π) can be calculated using Equation (7).

(7) $$\Pi =\left(1-\rho_{r}\right)*100$$

#### 4.3.4. Specific Surface Area

The specific surface area of the different samples was measured by nitrogen sorption through a Micrometrics (Norcross, GA, USA) ASAP 2020 instrument at the University of Málaga (Spain). Samples were degassed under vacuum for 24 h at 25 °C before starting the measurement. The experiment was performed at −196 °C and the specific surface area was calculated by the BET method [50] in the range of P/P_0_ = 0.05–0.35.

#### 4.3.5. Particle and Pore Size

Particle size was obtained through SEM (ESEM Scanning Electron Microscope QUANTA 200 FEG, Hillsboro, OR, USA) micrographs by measuring the individual particle contour of more than 50 individual particles with the Image J/FIJI software [51]. Since capillary condensation occurs when macropores are present in the nitrogen sorption experiments, Equation (8) is used to obtain a more accurate calculation of the pore volume:

(8) $$V_{p}=\frac{1}{\rho_{B}}-\frac{1}{\rho_{s}}$$

By assuming that pores in the aerogel have a cylindrical shape, pore size can be calculated with Equation (9) [50,52]:

(9) $$\Phi_{pore}=\frac{4V_{p}}{S_{BET}}$$

where V_p_ is the pore volume and S_BET_ is the specific surface area.

#### 4.3.6. Mechanical Tests

Compression tests were performed by a universal testing machine (Instron model 5500R6025), following the standard ASTM D1621-00 [53] at 23 ± 1 °C and 50 ± 5% of relative humidity as indicated by ISO 291:2008 [54]. All the samples were conditioned for 24 h prior to testing. The displacement rate was [height/10] mm/min and a load cell of 1 kN was used. The height of all the tested aerogels was ca. 20 mm.

Five load–unload cycles were performed at a strain of up to 10% to calculate the energy loss coefficient (ELC) from the hysteresis area [55]. This parameter was calculated following Equation (10):

(10) $$ELC\;\left(\%\right)=\frac{A_{L}-A_{U}}{A_{L}}\cdot 100$$

where A_L_ and A_U_ are the areas under the loading and the unloading cycle, respectively.

The elastic modulus (E) was also calculated from the slope of the linear region of the second compression–decompression curve. Once these cycles were completed, an additional compression cycle was performed until reaching a strain of ca. 80%.

#### 4.3.7. Thermal Conductivity Measurements

The thermal conductivity of the samples was measured by the steady-state method. These measurements were performed in a heat flow meter model FOX 314 (TA Instruments/LaserComp, Inc. New Castle, DE, USA) following the international normative ASTM C518 [56] and ISO 8301 [57]. The measurement method was modified according to the size of the samples (around 40 mm in diameter) by using an external sensor (gSKIN^®^ XM 27 9C, greenTEG AG) with dimensions of 10 × 10 × 0.5 mm^3^, which is placed on the lower side of the chamber, following a method previously developed and validated by Sánchez-Calderón et al. [58]. Thermal conductivity was measured at 20, 25, 30, and 40 °C, with a temperature gradient between plates of 20 °C.

#### 4.3.8. Sound Speed Tests

The speed of sound through the aerogels was measured by means of two non-focused air-coupled ultrasonic transducers (developed at ITEFI-CSIC), center frequency 500 kHz, −20 dB relative bandwidth of 75%, and 25 mm of aperture in transmission mode. The aerogel is placed between the transducers (one acts as transmitter: Tx, the other as receiver: Rx) at normal incidence. The transmitter transducer (Tx) is excited using a Panametrics 5077 pulser-receiver (P/R), by a semi cycle of a square wave, amplitude—200 V, tuned at transducers center frequency, with pulse repetition frequency (PRF) set to 100 Hz. The received signal in the receiver transducer (Rx) l is connected to the receiver stage of the P/R, amplified (up to 40 dB, when the aerogel is located between Tx and Rx), and, finally, transferred to a digital oscilloscope (Tektronix 5054) to be digitalized and stored.

Ultrasound velocity in the aerogel (ν) is obtained from the velocity in the air (ν_air_), the sample thickness (d), and the difference in time of flight when the sample is placed between transducers (∆t), as indicated in Equation (11).

(11) $$v_{aerogel}=\frac{d}{∆t+(\frac{d}{\nu_{air}})}$$

where the difference in the time of flight can be calculated from the edge detection or the maximum in the cross-correlation between both signals and the phase difference.

## Figures and Tables

**Table 1:** Gelation time of the opacified aerogels.

Gelation Time	L-GNP	M-GNP	S-GNP
**0.5 wt.%**	1′50″	3′01″	5′10″
**1 wt.%**	1′35″	3′33″	3′40″
**5 wt.%**	3′30″	2′30″	2′30″

**Table 2:** Main characteristics of the opacified aerogels.

Sample	ρ_B_ (kg/m^3^)	ρ_r_	S_l_ (%)	S_v_ (%)	Π (%)	S_BET_ (m^2^/g)	V_p_ (cm^3^/g)	Pore Size (nm)	Particle Size (nm)
**Reference**	78.47	0.0613	9.73	17.41	93.87	226.24	11.96	211.51	25.9 ± 3.7
**0.5 L**	80.44	0.0628	8.65	19.84	93.72	220.10	11.65	211.73	30.5 ± 5.0
**1 L**	82.99	0.0648	7.68	22.21	93.52	214.27	11.27	210.37	30.7 ± 4.5
**5 L**	87.57	0.0684	9.27	21.44	93.16	214.95	10.64	197.96	54.3 ± 7.7
**0.5 M**	96.85	0.0757	13.29	29.40	92.43	269.77	9.54	141.51	27.8 ± 4.3
**1 M**	98.34	0.0768	13.26	30.84	92.32	272.54	9.39	137.77	24.7 ± 3.4
**5 M**	92.27	0.0721	9.34	32.32	92.79	230.90	10.06	174.22	33.4 ± 7.4
**0.5 S**	92.96	0.0721	13.32	31.57	92.79	234.56	10.06	173.74	39.5 ± 5.7
**1 S**	88.25	0.0689	8.99	23.23	93.11	204.72	10.55	206.19	46.7 ± 6.7
**5 S**	88.48	0.0691	9.96	23.76	93.09	192.65	10.52	218.44	40.2 ± 6.2

## Data Availability

The data presented in this study are available on request from the corresponding author.

## Supplementary Materials

The following supporting information can be downloaded at: https://www.mdpi.com/article/10.3390/gels11010044/s1, Figure S1: (a) FT-IR spectra of reference (dashed line) and some opacified samples and (b) magnification of the FT-IR spectra in the range of 2000–500 cm^−1^. [59,60]; Figure S2: SEM image of 5 L sample with lower magnification; Figure S3: SEM micrographs of all the samples of this work The thermal conductivity at different temperatures of all the samples is shown in Figure S4; Figure S4: Thermal conductivity of (a) L-GNPs, (b) M-GNPs, and (c) S-GNPs at different temperatures (20, 25, 30, and 40 °C); Table S1: Sound speed of all the aerogels studied.

## References

[1] Kistler S.S. (1931). Coherent Expanded Aerogels and Jellies. Nature.

[2] Aerogel Market Size, Share, Growth & Trends Repot. 2030. https://www.grandviewresearch.com/industry-analysis/aerogel-market.

[3] Patel R., Purohit N., Suthar A., Patel S. (2009). An Overview of Silica Aerogels. Int. J. ChemTech Res..

[4] Hrubesh L.W., Pekala R.W. (1994). Thermal properties of organic and inorganic aerogels. J. Mater. Res..

[5] Tom C., Sinha S., Joshi N., Pujala R.K. (2022). Tuning Aerogel Properties for Aerospace Applications. Aerospace Polymeric Materials.

[6] Lei Y., Hu Z., Cao B., Chen X., Song H. (2017). Enhancements of thermal insulation and mechanical property of silica aerogel monoliths by mixing graphene oxide. Mater. Chem. Phys..

[7] Lu X., Arduini-Schuster M.C., Kuhn J., Nilsson O., Fricke J., Pekala R.W. (1992). Thermal Conductivity of Monolithic Organic Aerogels. Science.

[8] Wang Y.-Y., Zhou Z.-H., Zhu J.-L., Sun W.-J., Yan D.-X., Dai K., Li Z.-M. (2021). Low-temperature carbonized carbon nanotube/cellulose aerogel for efficient microwave absorption. Compos. Part B Eng..

[9] Nguyen S.T., Feng J., Ng S.K., Wong J.P.W., Tan V.B.C., Duong H.M. (2014). Advanced thermal insulation and absorption properties of recycled cellulose aerogels. Colloids Surf. A Physicochem. Eng. Asp..

[10] Biesmans G., Randall D., Francais E., Perrut M. (1998). Polyurethane-based organic aerogels’ thermal performance. J. Non-Cryst. Solids.

[11] Rigacci A., Marechal J.C., Repoux M., Moreno M., Achard P. (2004). Preparation of polyurethane-based aerogels and xerogels for thermal superinsulation. J. Non-Cryst. Solids.

[12] Szycher M. (2013). Szycher’s Handbook of Polyurethanes.

[13] Burgaz E. (2019). Polyurethane Insulation Foams for Energy and Sustainability.

[14] Merillas B., Villafañe F., Rodríguez-Pérez M.Á. (2022). Super-Insulating Transparent Polyisocyanurate-Polyurethane Aerogels: Analysis of Thermal Conductivity and Mechanical Properties. Nanomaterials.

[15] Lu X., Caps R., Fricke J., Alviso C.T., Pekala R.W. (1995). Correlation between structure and thermal conductivity of organic aerogels. J. Non-Cryst. Solids.

[16] Alvarez-Lainez M., Rodriguez-Perez M.A., De Saja J.A. (2007). Thermal Conductivity of Open-Cell Polyolefin Foams. J. Polym. Sci. Part B Polym. Phys..

[17] Jones D., Brischke C. (2017). 2—Wood as bio-based building material. Performance of Bio-Based Building Materials.

[18] Glicksman L.R. (1991). Heat Transfer and Ageing of Cellular Foam Insulation. Cell. Polym..

[19] Liu J., Buahom P., Lu C., Yu H., Park C.B. (2022). Microscopic revelation of the solid–gas coupling and Knudsen effect on the thermal conductivity of silica aerogel with inter-connected pores. Sci. Rep..

[20] Hilyard N.C., Cunningham A. (1994). Low Density Cellular Plastics: Physical Basis of Behaviour.

[21] Wang X.-D., Sun D., Duan Y.-Y., Hu Z.-J. (2013). Radiative characteristics of opacifier-loaded silica aerogel composites. J. Non-Cryst. Solids.

[22] Zhu J., Ren H., Bi Y. (2018). Opacified graphene-doped silica aerogels with controllable thermal conductivity. J. Porous Mater..

[23] Lamy-Mendes A., Malfait W.J., Sadeghpour A., Girão A.V., Silva R.F., Durães L. (2021). Influence of 1D and 2D carbon nanostructures in silica-based aerogels. Carbon.

[24] Karamikamkar S., Abidli A., Behzadfar E., Rezaei S., Naguib H.E., Park C.B. (2019). The effect of graphene-nanoplatelets on gelation and structural integrity of a polyvinyltrimethoxysilane-based aerogel. RSC Adv..

[25] Hümmer E., Lu X., Rettelbach T., Fricke J. (1992). Heat transfer in opacified aerogel powders. J. Non-Cryst. Solids.

[26] Kuhn J., Gleissner T., Arduini-Schuster M.C., Korder S., Fricke J. (1995). Integration of mineral powders into SiO_2_ aerogels. J. Non-Cryst. Solids.

[27] Kwon Y.-G., Choi S.-Y., Kang E.-S., Baek S.-S. (2000). Ambient-dried silica aerogel doped with TiO_2_ powder for thermal insulation. J. Mater. Sci..

[28] Zhang H., Qiao Y., Zhang X., Fang S. (2010). Structural and thermal study of highly porous nanocomposite SiO_2_-based aerogels. J. Non-Cryst. Solids.

[29] Liu H.-L., He X., Li H.-Y., Li J., Li Y.-J. (2018). Novel GO/silica composite aerogels with enhanced mechanical and thermal insulation properties prepared at ambient pressure. Ferroelectrics.

[30] Lamy-Mendes A., Girão A.V., Silva R.F., Durães L. (2019). Polysilsesquioxane-based silica aerogel monoliths with embedded CNTs. Microporous Mesoporous Mater..

[31] Tafreshi O.A., Ghaffari-Mosanenzadeh S., Ben Rejeb Z., Saadatnia Z., Rastegardoost M.M., Zhang C., Park C.B., Naguib H.E. (2023). Amphiphilic polyimide-graphene nanoplatelet aerogel composites with high mechanical stability and enhanced thermal insulation properties for oil sorption applications. Mater. Today Sustain..

[32] Zhu C.Y., Li J.B., Dai P.C., Gong L. (2024). Elevating high-temperature insulation performance of silica aerogels enabled by innovative surface-structured opacifiers. Appl. Therm. Eng..

[33] Liu H., Liu J., Tian Y., Jiao J., Wu X. (2022). Thermal Insulation Performance of Silica Aerogel Composites Doped with Hollow Opacifiers: Theoretical Approach. Gels.

[34] Deng T., Li H., Li Y., Jiang C., He Y., Yang T., Zhu L., Xie L. (2023). Environment friendly biomass composite aerogel with reinforced mechanical properties for thermal insulation and flame retardancy application. Polym. Eng. Sci..

[35] Merillas B., Álvarez-Arenas T.E.G., Villafañe F., Angel Rodríguez-Pérez M. (2023). Reaching a near zero radiative heat transfer by the inclusion of modified multiwalled-carbon nanotubes (MWCNTs) in polyurethane-polyisocyanurate aerogels. Mater. Today Chem..

[36] Martín-de León J., Sillero A., Rodríguez-Pérez M.A. (2024). Using infrared opacifiers to reduce the thermal conductivity of micro and nanocellular polymethylmethacrylate. Polymer.

[37] Wolf A., Terheiden B., Brendel R. (2008). Light scattering and diffuse light propagation in sintered porous silicon. J. Appl. Phys..

[38] Merillas B., Martín-De León J., Villafañe F., Ángel Rodríguez-Pérez M. (2022). Optical Properties of Polyisocyanurate-Polyurethane Aerogels: Study of the Scattering Mechanisms. Nanomaterials.

[39] Merillas B., Martín-De León J., Villafañe F., Rodríguez-Pérez M.A. (2021). Transparent Polyisocyanurate-Polyurethane-Based Aerogels: Key Aspects on the Synthesis and Their Porous Structures. ACS Appl. Polym. Mater..

[40] Notario B., Pinto J., Solorzano E., de Saja J.A., Dumon M., Rodríguez-Pérez M.A. (2015). Experimental validation of the Knudsen effect in nanocellular polymeric foams. Polymer.

[41] Forest C., Chaumont P., Cassagnau P., Swoboda B., Sonntag P. (2015). Polymer nano-foams for insulating applications prepared from CO_2_ foaming. Prog. Polym. Sci..

[42] Zhao J.-J., Duan Y.-Y., Wang X.-D., Zhang X.-R., Han Y.-H., Gao Y.-B., Lv Z.-H., Yu H.-T., Wang B.-X. (2013). Optical and radiative properties of infrared opacifier particles loaded in silica aerogels for high temperature thermal insulation. Int. J. Therm. Sci..

[43] Wang L., Wu Y.K., Ai F.F., Fan J., Xia Z.P., Liu Y. (2018). Hierarchical porous polyamide 6 by solution foaming: Synthesis, characterization and properties. Polymers.

[44] Zhao C., Mark L.H., Chang E., Chu R.K.M., Lee P.C., Park C.B. (2020). Highly expanded, highly insulating polypropylene/polybutylene-terephthalate composite foams manufactured by nano-fibrillation technology. Mater. Des..

[45] Silva M.C., Takahashi J.A., Chaussy D., Belgacem M.N., Silva G.G. (2010). Composites of rigid polyurethane foam and cellulose fiber residue. J. Appl. Polym. Sci..

[46] Jelle B.P. (2011). Traditional, state-of-the-art and future thermal building insulation materials and solutions—Properties, requirements and possibilities. Energy Build..

[47] Zhao J., Wang G., Wang C., Park C.B. (2020). Ultra-lightweight, super thermal-insulation and strong PP/CNT microcellular foams. Compos. Sci. Technol..

[48] Koebel M., Rigacci A., Achard P. (2012). Aerogel-based thermal superinsulation: An overview. J. Sol-Gel Sci. Technol..

[49] Standard Test Method for Apparent Density of Rigid Cellular Plastics.

[50] Barrett E.P., Joyner L.G., Halenda P.P. (1951). The Determination of Pore Volume and Area Distributions in Porous Substances. I. Computations from Nitrogen Isotherms. J. Am. Chem. Soc..

[51] Pinto J., Solórzano E., Rodriguez-Perez M.A., de Saja J.A. (2013). Characterization of the cellular structure based on user-interactive image analysis procedures. J. Cell. Plast..

[52] Horvat G., Pantić M., Knez Ž., Novak Z. (2022). A Brief Evaluation of Pore Structure Determination for Bioaerogels. Gels.

[53] Standard Test Method for Compressive Properties of Rigid Cellular Plastics.

[54] Plastics—Standard Atmospheres for Conditioning and Testing.

[55] Bhinder J., Agnihotri P.K. (2020). Effect of carbon nanotube doping on the energy dissipation and rate dependent deformation behavior of polyurethane foams. J. Cell. Plast..

[56] (2021). Standard Test Method for Steady-State Thermal Transmission Properties by Means of the Heat Flow Meter Apparatus 1.

[57] Thermal Insulation—Determination of Steady-State Thermal Resistance and Related Properties—Heat Flow Meter Apparatus—Amendment 1.

[58] Sánchez-Calderón I., Sillero A., Lizalde-Arroyo F., Bernardo V., Martín-De-León J., Angel Rodríguez-Pérez M. (2023). Evaluation of methods to accurately characterize the thermal conductivity of micro-and nanocellular polymers based on poly(methyl-methacrylate) (PMMA) produced at lab-scale. Polym. Test..

[59] Rewatkar P.M., Saeed A.M., Far H.M., Donthula S., Sotiriou-Leventis C., Leventis N. (2019). Polyurethane Aerogels Based on Cyclodextrins: High-Capacity Desiccants Regenerated at Room Temperature by Reducing the Relative Humidity of the Environment. ACS Appl. Mater. Interfaces.

[60] Romero R.R., Grigsby R.A., Rister E.L., Pratt J.K., Ridgway D. (2005). A Study of the Reaction Kinetics of Polyisocyanurate Foam Formulations using Real-time FTIR. J. Cell. Plast..

