# Supplementary material for: Studying the Size-Dependence of Graphene Nanoplatelets (GNPs) in the Final Properties of Polyurethane Aerogels: Thermal Insulation and Mechanical Strength
Source: Gels. 2025 Jan 7;11(1):44. doi: 10.3390/gels11010044 (PMC11764653; doi:10.3390/gels11010044)
Supplement: Supplementary file 1 [file gels-11-00044-s001.zip › gels-3396059-supplementary-done.pdf]

gels

MDPI

Article

Studying the Size-Dependence of Graphene Nanoplatelets (GNPs) in the Final Properties of Polyurethane Aerogels: Thermal Insulation and Mechanical Strength

Jaime Lledó [1,2], Judith Martín-de León [1,2], Tomás E. Gómez Álvarez-Arenas [3], Miguel Ángel Rodríguez-Pérez [1,2] and Beatriz Merillas [1,2,4,*]

[1] Cellular Materials Laboratory (CellMat), Condensed Matter Physics Department, Faculty of Science, Campus Miguel Delibes, University of Valladolid, Paseo de Belén 7, 47011 Valladolid, Spain; jaime.lledo@uva.es (J.L.); judit.martin.leon@uva.es (J.M.-d.L.); marrod@uva.es (M.Á.R.-P.)
[2] BioEcoUVA Research Institute on Bioeconomy, University of Valladolid, Spain
[3] Departamento de Sensores y Sistemas Ultrasónicos (DSSU), Instituto de Tecnologías Físicas y de la Información (ITEFI), Consejo Superior de Investigaciones Científicas (CSIC), Serrano 144, 28006 Madrid, Spain; t.gomez@csic.es (T.E.G-A-A)
[4] Department of Chemical Engineering, CERES, University of Coimbra, Rua Sílvio Lima, 3030-790 Coimbra, Portugal
* Correspondence: beatriz.merillas@uva.es

Figure S1 shows the FT-IR spectra of all the samples.

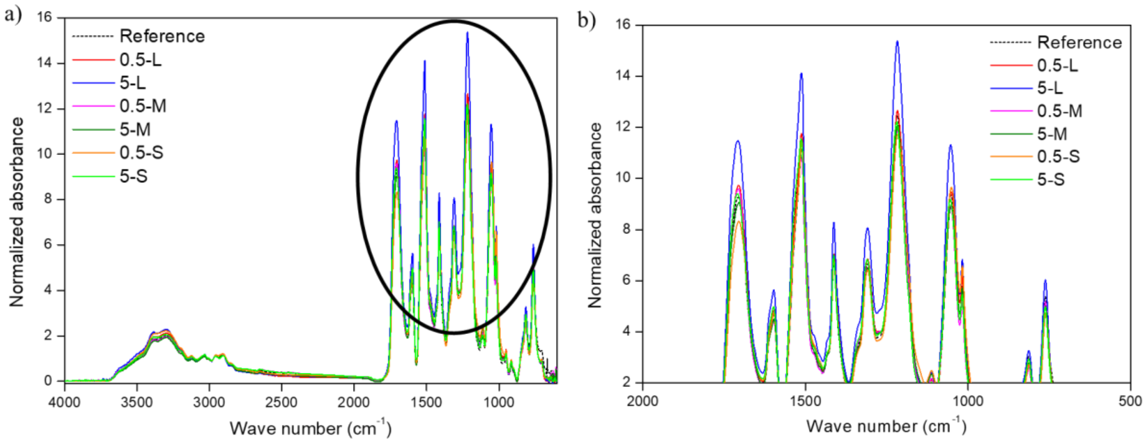

Figure S1. a) FT-IR spectra of reference (dashed line) and some opacified samples and b) magnification of the FT-IR spectra in the range of 2000-500 cm^{-1}.

The FTIR spectra of the final aerogels reveal the characteristic peaks of polyurethane and polyisocyanurate. It is observed a broad absorption band in the range of 3600-3200 cm^{-1}, corresponding to the N-H stretching [59]. Moreover, urethane and urea carbonyl stretches are observed in 1709 and 1597 cm^{-1}, respectively. The N-H bending coupled to the C-N stretching is observed at 1513 cm^{-1}. Finally, the isocyanurate C-N stretching is present at 1412 cm^{-1} [60] and the C-N stretch can also be observed at 1217 cm^{-1}. The FTIR spectra show that the peak area increases (Figure S1b) with the particle addition when normalizing at the asymmetric -CH stretching band at 2972 cm^{-1} [14]. It is also revealed that there is no chemical bonding between GNPs and the matrix since the peaks from the IR spectra do not change when adding these fillers, so it can be deduced that these fillers

 https://doi.org/10.3390/gels11010044

are embedded in the aerogel structure, and chains of polyurethane–polyisocyanurate chains are physically surrounding these particles.

In Figure S2 a SEM image of a 5 L sample with a lower magnification is shown. Note that individual particles are present, not showing any indication of the presence of aggregates.

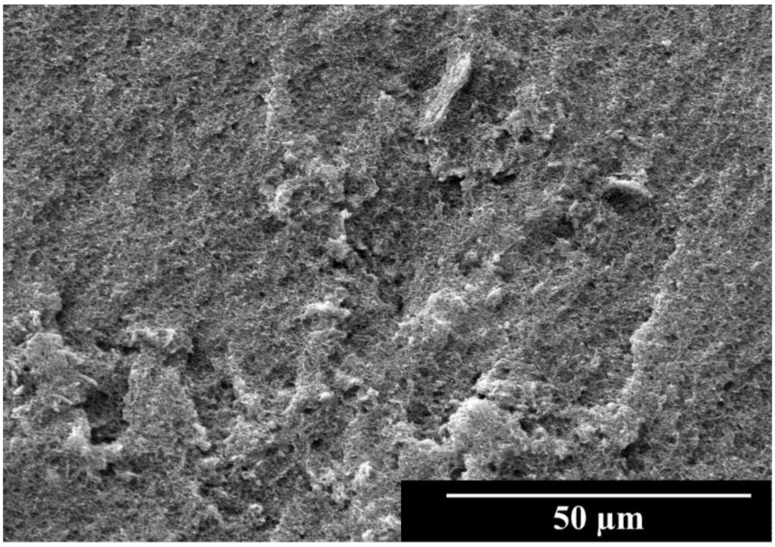

Figure S2. SEM image of 5 L sample with lower magnification.

SEM micrographs of all the synthesized samples are shown in Figure S3. All samples present a homogeneous structure. It is also discernible how L and S-GNP samples present a higher pore size, comparable to the reference sample, than M-GNP aerogels.

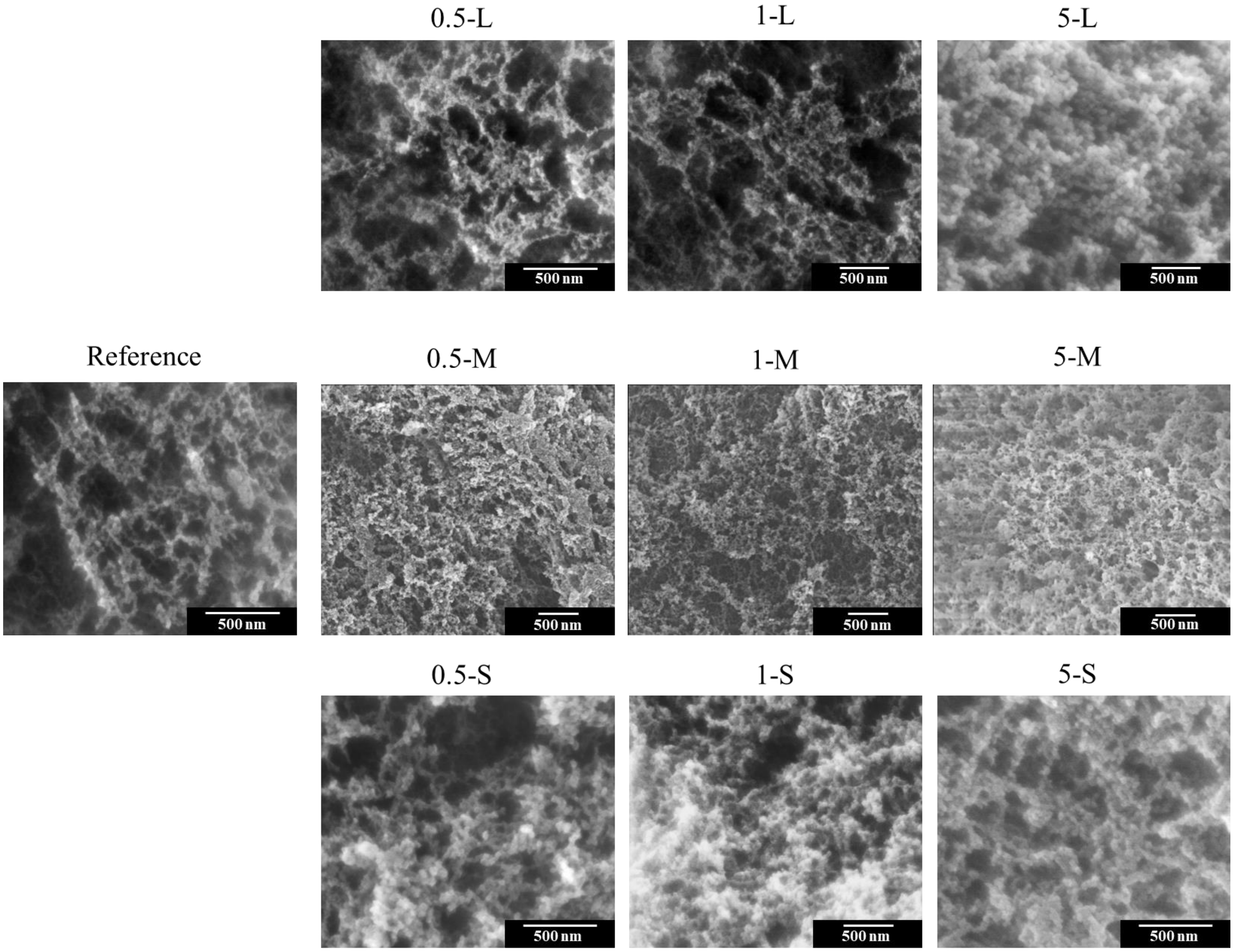

Figure S3. SEM micrographs of all the samples of this work The thermal conductivity at different temperatures of all the samples is shown in Figure S.4.

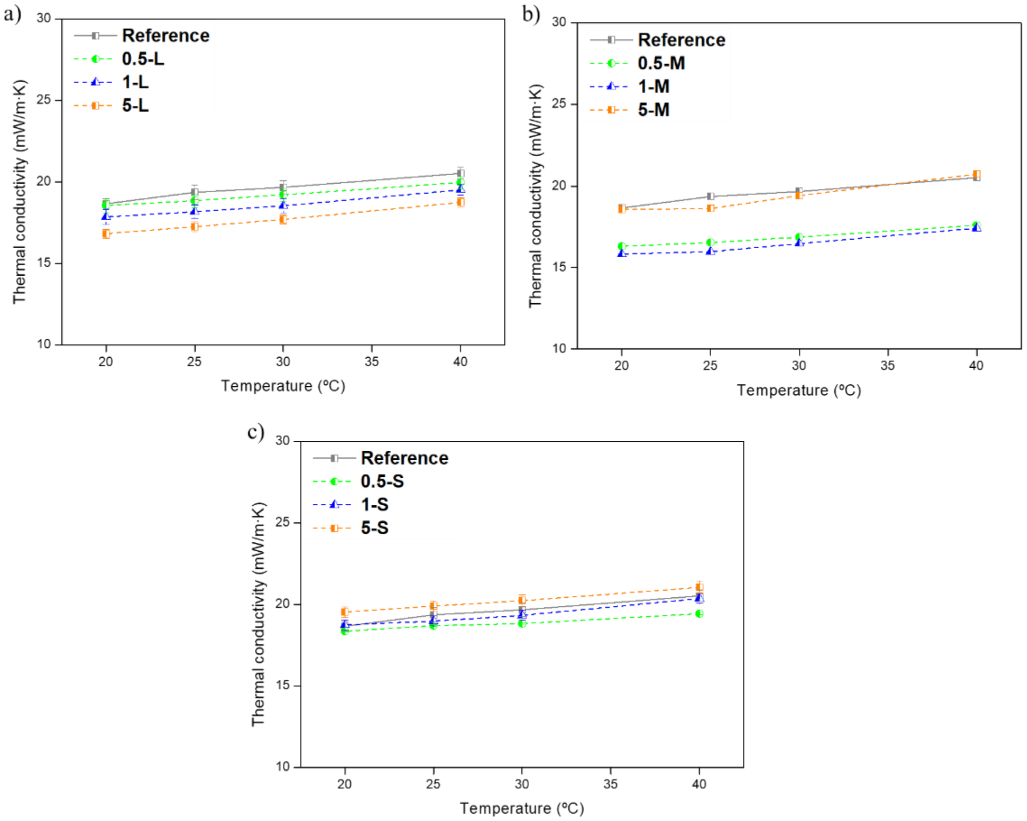

Figure S4. Thermal conductivity of a) L-GNPs, b) M-GNPs, and c) S-GNPs at different temperatures (20, 25, 30, and 40 ºC).

In Table S1 the sound speed used for calculating the conductivity through the solid phase can be found. It is observed that the sound speeds follow the same trend as solid thermal conduction. The largest sound speeds correspond to the samples with higher solid thermal conduction (S-GNP series), whereas lower sound speeds correspond to lower solid thermal conduction, as observed for L and M-GNP.

Table S1. Sound speed of all the aerogels studied.

Sample	Sound speed (m/s)
**Reference**	105.6
**0.5 L**	95.0
**1 L**	95.0
**5 L**	126.0
**0.5 M**	109.2
**1 M**	109.0
**5 M**	109.0
**0.5 S**	152.0
**1 S**	139.0
**5 S**	134.8

References

14. B. Merillas, F. Villafañe, and M. Á. Rodríguez-Pérez, "Super-Insulating Transparent Polyisocyanurate-Polyurethane Aerogels: Analysis of Thermal Conductivity and Mechanical Properties," *Nanomaterials*, vol. 12, no. 14, p. 2409, Jul. 2022, doi: 10.3390/NANO12142409/S1.
59. P. M. Rewatkar, A. M. Saeed, H. M. Far, S. Donthula, C. Sotiriou-Leventis, and N. Leventis, "Polyurethane Aerogels Based on Cyclodextrins: High-Capacity Desiccants Regenerated at Room Temperature by Reducing the Relative Humidity of the Environment," 2019, doi: 10.1021/acsami.9b10755.
60. R. R. Romero, R. A. Grigsby, E. L. Rister, J. K. Pratt, and D. Ridgway, "A Study of the Reaction Kinetics of Polyisocyanurate Foam Formulations using Real-time FTIR*," 2005, doi: 10.1177/0021955X05055115.

Disclaimer/Publisher's Note: The statements, opinions and data contained in all publications are solely those of the individual author(s) and contributor(s) and not of MDPI and/or the editor(s). MDPI and/or the editor(s) disclaim responsibility for any injury to people or property resulting from any ideas, methods, instructions or products referred to in the content.
